# Exploring the Prognostic Value of Tumour‐Associated Genes in Clear Cell Renal Cell Carcinoma Through Single‐Cell RNA Sequencing Insights

**DOI:** 10.1111/jcmm.70297

**Published:** 2024-12-20

**Authors:** Tongfei Fu, Xinyi Zhang, Yuedong Liu, Junsong Wu, Xuefeng Liu, Bichao Lu, Yi Huang, Liping Yang, Yongli Zhan

**Affiliations:** ^1^ Department of Nephrology, Guang'anmen Hospital China Academy of Chinese Medical Sciences Beijing China; ^2^ The First Clinical College Hubei University of Chinese Medicine Wuhan China; ^3^ Department of Anorectal The Third Affiliated Hospital of Liaoning University of Traditional Chinese Medicine ShenYang China; ^4^ The Third Clinical Department Liaoning University of Traditional Chinese Medicine ShenYang China; ^5^ College of Clinical Chinese Medicine Hubei University of Chinese Medicine Wuhan China; ^6^ Department of Critical Care Medicine Yichang Hospital of Traditional Chinese Medicine Yichang China

**Keywords:** CCL2, ccRCC, endothelial cell function, notch, prognostic model

## Abstract

Clear cell renal cell carcinoma (ccRCC) characterised by its diversity and a tendency to defy standard therapeutic approaches. Amidst the advent of immunotherapy, it has become imperative to pinpoint prognostic indicators of the tumour microenvironment (TME) influence the efficacy of treatments. Employing single‐cell RNA sequencing (scRNA‐seq), this research delved into the diverse landscape of ccRCC, uncovering its complex underpinnings and pinpointing molecular avenues for therapeutic intervention. We constructed a prognostic model using 101 machine learning algorithms and integrated data from multiple cohorts, including TCGA, ICGC, and microarray datasets. The model's efficacy was assessed using the Concordance Index (C‐index), and further analyses included pseudotime analysis of tumour cells, mutation analysis and correlation analysis between the prognostic model and tumour immunity. The prognostic model, combining Lasso regression and survival Support Vector Machine (SVM), demonstrated robust discrimination with a C‐index of 0.650. Investigation into the TME uncovered pronounced associations between the presence of immune cell infiltrates and patient outcomes, with a notable emphasis on the impact of CCL2‐expressing neoplastic cells. The GO Biological Processes (GOBP) encompass the regulation of endothelial cell maturation, the formation of endothelial layers, the enhancement of gene expression controlled by Notch receptors, and the development of endothelial barriers. The research effectively pinpointed critical prognostic markers and crafted a forecasting model that achieved a C‐index of 0.650, highlighting the significant impact of immune cell infiltration, especially CCL2+ neoplastic cells, on ccRCC patient prognosis.

## 1 Background

Globally, renal cell carcinoma (RCC) stands as the 14th leading cancer in terms of new diagnoses, with over 400,000 individuals affected in 2020 [1]. It ranks among the most lethal malignancies of the urinary tract, exhibiting a rising incidence annually [2]. The clear cell variant of RCC, known as clear cell renal cell carcinoma (ccRCC), predominates this category, constituting approximately 70%–85% of RCC diagnoses [3, 4]. Although the specific incidence and mortality rates may vary across different regions, ccRCC is generally considered a relatively fatal cancer type. ccRCC significantly impacts patients' quality of life, as this cancer can lead to renal failure, pain, and other complications requiring long‐term treatment and monitoring. Around one in five individuals with late‐stage RCC possess tumours characterised by the presence of sarcomatoid features [5]. These sarcomatoid‐attributed RCC tumours exhibit an exceptionally aggressive nature, which often results in swift metastatic spread and unfavourable treatment outcomes [6].

The challenges in treating ccRCC include the heterogeneity of the disease and resistance to traditional treatment methods. Furthermore, the treatment of ccRCC often involves surgery, radiation therapy, and drug therapy, which can potentially lead to side effects that impact patients' quality of life [1, 7].

The ecosystem of cells and substances surrounding the tumour is crucial in the development, functioning, and therapeutic intervention of RCC. Specifically, ccRCC, the predominant form of RCC, often includes distinctive secondary inactivation mutations within the VHL gene, typically against a backdrop of 3p chromosome deletions [8, 9]. Such genetic alterations result in the stabilisation of hypoxia‐inducible factors (HIFs), prompting affected cells to resort to glycolysis and to emit VEGF, a key driver of new blood vessel formation [10]. Additionally, ccRCC is notably permeated by immune cells, with a significant presence of T cells [11]. Capitalising on these microenvironmental traits, therapeutic strategies have markedly progressed in the past 10 years, featuring extensive application of VEGF tyrosine kinase inhibitors (TKIs), immune checkpoint blockade (ICB), and their combined modalities [12, 13, 14].

Nevertheless, foreseeing treatment responsiveness among patients continues to be a core dilemma. Evidence suggests that genomic indicators, which show a correlation with ICB efficacy in other solid tumours—like tumour mutational burden (TMB) and PD‐L1 expression levels [15, 16, 17], fail to forecast therapeutic success in ccRCC [18, 19]. This inconsistency implies that the tumour's immune contexture could play a substantial part in determining the therapeutic payoff. Supporting this notion, various studies have established ccRCC as a cancer type characterised by pronounced immune and vascular invasion [11, 20, 21].

The genomic characterisation of responses to ICB has primarily been conducted on bulk‐sequenced pre‐treatment tumour samples. Within the scope of these investigations, a reduced initial presence of myeloid inflammation and the occurrence of non‐functional mutations within the PBRM1 gene have been linked to positive treatment outcomes within certain patient groups [18, 19, 22]. The presence of both intra‐ and inter‐tumoral heterogeneity poses challenges for a mechanistic understanding of ICB response. ccRCC presents a spectrum of molecular subtypes, characterised by alterations in a set of genes that are pivotal for chromatin remodelling. These subtypes display distinct patterns of tumour evolution and metastatic behaviour [23, 24].

Historically, genomic studies of ccRCC have concentrated on analysing patients who have not yet received ICB therapy, utilising techniques such as bulk RNA sequencing (RNA‐seq) [11] and mass cytometry [25]. However, these methodologies are inherently limited in their ability to capture the full spectrum of the immune microenvironment due to their resolution constraints. To enhance the precision of patient response forecasting and to facilitate the advancement of tailored therapeutic strategies, a more detailed and high‐resolution mapping of the ccRCC immune landscape is imperative.

Prior research in the field of transcriptomics has predominantly concentrated on biopsies from single tumour regions, potentially leading to an underestimation of the diversity in immune cell infiltration across different patient samples [26]. In this study, we utilised transcriptomic data, combined with single‐cell RNA sequencing and machine learning to establish a prognostic model, thereby comprehensively dissecting the prognostic roles of genes associated with immune therapy‐related tumours in ccRCC.

## 2 Method

### 2.1 Compilation and Examination of Genome‐Wide Expression Profiles

For the purpose of model development, RNA expression datasets and their clinical correlates (*n* = 531) from ccRCC cases within TCGA repository were procured. A separate cohort of RNAseq data (*n* = 91) from the ICGC was utilised to validate the model's reliability and precision. The data were normalised according to the Transcripts Per Million (TPM) scale and subsequently processed through a log^2^ transformation in preparation for advanced analytical procedures. In addition, microarray datasets (E‐MTAB‐1980, GSE167573 and GSE29609) encompassing 196 ccRCC cases from the GEO and EMBL databases were incorporated as an independent validation cohort. Microarray data normalisation was carried out utilising the limma package's normalizeBetweenArrays function, and batch effect interference was reduced by employing the Combat function from the sva package.

### 2.2 Compilation and Optimization of Single‐Cell RNA Sequencing Data for Comprehensive Tumour Analysis

We sourced our single‐cell RNA sequencing datasets from the Gene Expression Omnibus (GEO) database, specifically focusing on GSE152938 and GSE237425. The GSE152938 cohort comprised four tumour samples, encompassing two ccRCCs, one chromophobe renal cell carcinoma, and one papillary renal cell carcinoma, all from patients under the age of 50. Additionally, we included four additional ccRCC samples from patients over 60 years old, sourced from GSE237425, totalling eight samples for our study. To ensure rigorous data analysis, we leveraged R software (version 4.1.3) in conjunction with the Seurat package. Our quality control criteria stipulated stringent thresholds for mitochondrial and haematopoietic cell content, maintaining them below 30% and 3%, respectively. Furthermore, we imposed constraints on the counts of unique molecular identifiers (UMIs) and genes, ensuring they fell within the ranges of 200–30,000 and 200–7000, respectively. Following quality control, we embarked on data normalisation, selecting 2000 highly variable genes for further analysis. To account for cell cycle effects, we utilised the NormalizeData, FindVariableFeatures, and ScaleData functions from Seurat. To mitigate potential batch effects, we employed the Harmony algorithm, ensuring the integrity of our cross‐dataset comparisons. Subsequently, we implemented dimensionality reduction using the Uniform Manifold Approximation and Projection (UMAP) method, facilitated by Seurat. This step facilitated the visualisation of complex datasets in a lower‐dimensional space, preserving important structural information. For clustering, we adopted the Louvain method, which efficiently partitioned our cells into distinct groups based on their transcriptional profiles. Finally, to discern differentially expressed genes among clusters or cell types, we utilised the FindAllMarkers function, applying rigorous statistical thresholds of a *p* value < 0.05, log2 fold change > 0.25, and a minimum expression proportion of 0.1. Data from empty droplets in GSE210041 from GEO were processed and visualised using Seurat, with cell type annotation facilitated by the CARD software.

### 2.3 Cell Annotation Analysis

Markers indicative of fibroblasts (“COL1A1”, “COL1A2”, “DCN”, “THY1”), endothelial cells (“FLT1”, “RAMP2”, “PECAM1”, “CLDN5”), T cells (“CD3G”, “TRAC”, “CD3D”, “CD3E”), NK cells (“NCAM1”, “KLRD1”, “NKG7”, “GNLY”), B cells (“IGHG3”, “IGHA2”, “CD79A”, “IGHM”), myeloid cells (“FCGR3A”, “LYZ”, “MARCO”, “CD68”), and mast cells (“GATA2”, “KIT”, “MS4A2”) were identified. Epithelial cell markers (“EPCAM”, “KRT18”, “KRT19”, “CDH1”) were also used. Subsequently, specialised single‐cell analytical software was applied to classify the subtypes of immune cells and to produce visual representations, including UMAP plots, bar charts, and heatmaps.

### 2.4 Identification of Prognostic Genes

Univariate Cox analysis was performed on all genes across TCGA and two validation sets (ICGC, microarray) to select 11 prognostic genes (*p* < 0.05 in at least three datasets). The remaining datasets served as validation for PIS model construction with these 11 genes.

### 2.5 Establishment of Tumour‐Related Risk Signatures

An extensive ensemble of 101 machine learning algorithms was deployed to devise predictive models tailored to generate individualised risk scores for patients. These models leveraged the sophistication of modern machine learning techniques to capture intricate patterns in patient data. Subsequently, the surv_cutpoint function was meticulously applied to ascertain optimal threshold values, enabling the stratification of patients into distinct high‐ and low‐risk groups. This stratification process was rigorously carried out across the comprehensive TCGA dataset, as well as additional study populations. The efficacy of the models was gauged by examining the differential predictive outcomes between the risk groups and by evaluating the precision of the predictions.

### 2.6 Creation of a Composite Risk Signature Utilising Machine Learning Techniques

To refine the Prognostic Index Score (PIS) model, we integrated 10 machine learning algorithms (RSF, Enet, Lasso, Ridge, Stepwise Cox, CoxBoost, plsRcox, SuperPC, GBM, survival‐SVM) and explored 101 algorithmic combinations, aiming for increased precision and reliability. The process of crafting the risk signature encompassed several steps: (a) the identification of predictive biomarkers through univariate Cox regression analysis across three datasets, including TCGA; (b) the construction of predictive models employing leave‐one‐out cross‐validation (LOOCV) within the TCGA‐KIRC cohort, leveraging the 101 algorithmic combinations; (c) the verification of all candidate models across two distinct validation datasets, namely ICGC and microarray data; (d) the selection of the optimal model, determined by the highest mean Harrell's Concordance Index (C‐index) across the validation datasets.

### 2.7 Pseudotime Analysis of Single Cells

Monocle2 software was used for pseudotime analysis of tumour cell subpopulations, with DDRTree for dimensionality reduction and default parameters for other settings, to infer cell differentiation processes. CytoTRACE software further explored tumour cell differentiation levels.

### 2.8 Mutation Analysis

GISTIC 2.0 software analysed Copy Number Variations (CNVs) in patients, and maftools software calculated Tumour Mutation Burden (TMB).

### 2.9 Correlation Analysis Between Prognostic Model and Tumour Immunity

IOBR software determined immune infiltration levels in TCGA patients, incorporating results from seven assessment methods. Heatmap visualisations depicted the comparative distribution of immune cell infiltration throughout the TME. The “estimate” R package was employed to evaluate the relative frequencies of stromal, immune and tumour cell populations, with the results being analysed and contrasted across varying risk groups.

### 2.10 Prediction of Tumour Composition in Bulk Data

The BisqueRNA R package predicted the composition of tumour subpopulations in bulk datasets based on single‐cell data, while GSVA algorithm calculated scores for bulk tumour subpopulations using subpopulation markers.

### 2.11 Prediction of Immunotherapy Response

The TIDE algorithm predicted immunotherapy responses in RNAseq (TCGA+ICGC) and microarray datasets. GSE78220 (melanoma), GSE93157 (melanoma), GSE67501 (RCC), PRJEB25780 (STAD), PRJNA482620 (GBM) and GSE93157 (LUSC) datasets were used to calculate PIS scores and evaluate PIS differences in immunotherapy response.

### 2.12 Execution of Statistical Evaluations

The entirety of data manipulation, statistical computations, and graphical representation was accomplished using R version 4.1.3. The Pearson correlation coefficient was deployed to measure the strength of association between continuous variables. For categorical variables, chi‐square tests were applied to identify significant differences, while the Wilcoxon rank‐sum or *t*‐tests were used to make comparisons among continuous variables. The survminer package was instrumental in identifying the most suitable cut‐off thresholds. The survival package facilitated the execution of Cox regression and Kaplan–Meier survival analysis.

### 2.13 Cell Culture

The human renal proximal tubular epithelial cell line HK‐2 and the human renal cell carcinoma cell line 786‐O were obtained from the American Type Culture Collection (ATCC, USA). Both cell lines were authenticated prior to use to ensure validity and purity. HK‐2 cells were cultured in Dulbecco's Modified Eagle Medium/F12 (DMEM/F12; Gibco, USA) supplemented with 10% fetal bovine serum (FBS; Gibco, USA) and 1% penicillin–streptomycin (Pen‐Strep; Gibco, USA). The cells were maintained at 37°C in a humidified atmosphere containing 5% CO₂. 786‐O cells were maintained in RPMI 1640 medium (Gibco, USA) supplemented with 10% FBS and 1% Pen‐Strep. The incubation conditions were the same as for HK‐2 cells, with the temperature set at 37°C and 5% CO₂. Both cell lines were passaged at approximately 80% confluence, and medium was replaced every 2–3 days. Cells in the logarithmic growth phase were used for all experiments.

### 2.14 Real‐Time Quantitative Polymerase Chain Reaction

Total RNA was extracted from cells using the TRIzol reagent (Invitrogen, USA) following the manufacturer's protocol. The quality and quantity of RNA were assessed using a NanoDrop spectrophotometer (Thermo Fisher Scientific, USA). Subsequently, 1 μg of RNA was reverse transcribed into complementary DNA (cDNA) using the PrimeScript RT Master Mix (Takara, Japan) according to the manufacturer's instructions.

qPCR was performed using the TB Green Premix Ex Taq II (Takara, Japan) on a QuantStudio five Real‐Time PCR System (Applied Biosystems, USA). The reaction conditions included an initial denaturation step at 95°C for 30 s, followed by 40 cycles of 95°C for 5 s and 60°C for 30 s. The primer sequences for target genes and the housekeeping gene were designed and synthesised by Sangon Biotech (China). Relative gene expression levels were calculated using the 2^−ΔΔCt method, normalising the expression of target genes to GAPDH. All reactions were performed in triplicate to ensure accuracy and reproducibility. Primer sequences are listed in Table S1.

## 3 Result

### 3.1 Comprehensive Single‐Cell Landscape of ccRCC

**FIGURE 1:**
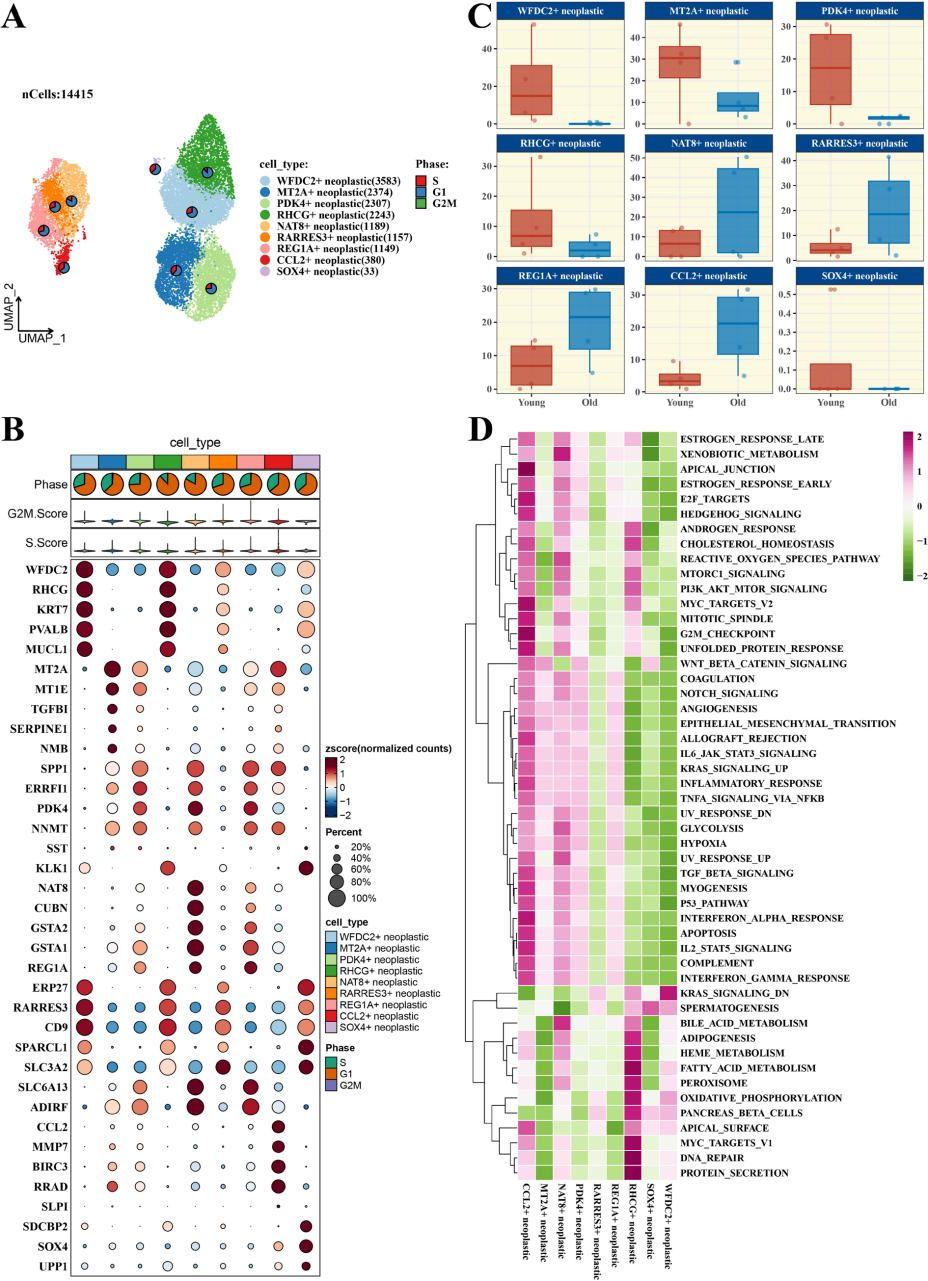
Comprehensive Single‐Cell Profiling of RCC. (A) UMAP plot coloured by cell types derived from single‐cell data. (B) Bubble chart depicting the expression patterns of signature genes across the spectrum of tumour cell subsets. (C) Boxplot showing the difference in the proportion of tumour cell subpopulations among different patient age groups. (D) Heatmap of ssGSEA scores for 50 Hallmark pathways associated with each tumour cell subpopulation.

Within the scope of this research, the UMAP algorithm was utilised to execute dimensionality reduction and to cluster the cells within ccRCC tumours (refer to Figure S1). Figure 1A illustrates the spatial distribution of the diverse cell types. Subsequent analyses were carried out to probe the expression of marker genes within distinct tumour cell subpopulations, with the results presented in a bubble plot format (Figure 1B). A comparative examination of the distribution of tumour cell subpopulations across different age groups of patients was undertaken (Figure 1C), and single‐sample Gene Set Enrichment Analysis (ssGSEA) scores pertaining to 50 Hallmark gene sets were determined (Figure 1D), thereby shedding light on the intrinsic biological properties of the tumour cells.

### 3.2 Pseudotime Analysis of Tumour Cells

**FIGURE 2:**
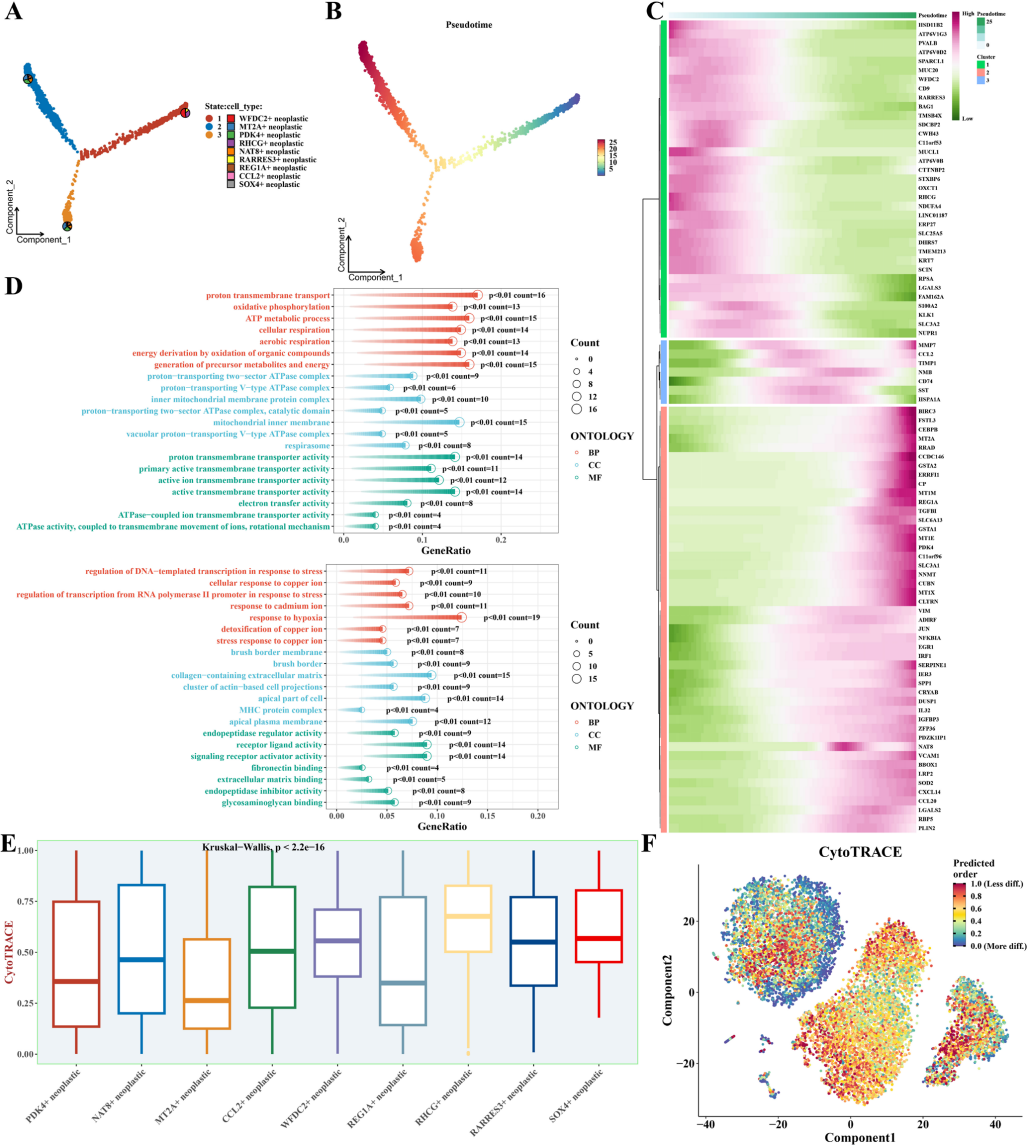
Trajectory Analysis of Tumour Cell Evolution. (A, B) Outcomes from the Monocle algorithm applied to tumour cell progression. (C) A heatmap delineating genes correlated with pseudotime alongside markers characteristic of each tumour cell subset. (D) Gene Ontology (GO) enrichment findings for genes categorised as C1 and the C2‐3 groupings. (E, F) CytoTRACE software‐derived assessments of the differentiation trajectory of tumour cells.

Pseudotime analysis of tumour cells using monocle software revealed changes in cellular states during tumour development (Figure 2A,B). By identifying genes correlated with pseudotime and sorting them into three distinct categories—C1, C2 and C3—functional assessments of these genes were conducted employing GO enrichment analysis, as depicted in Figure 2C,D. Additionally, CytoTRACE software was utilised to analyse the differentiation degree of tumour cells, revealing significant differences among distinct subpopulations (Figure 2E,F).

### 3.3 Integrative Analysis of Single‐Cell and Immune Cohorts

**FIGURE 3:**
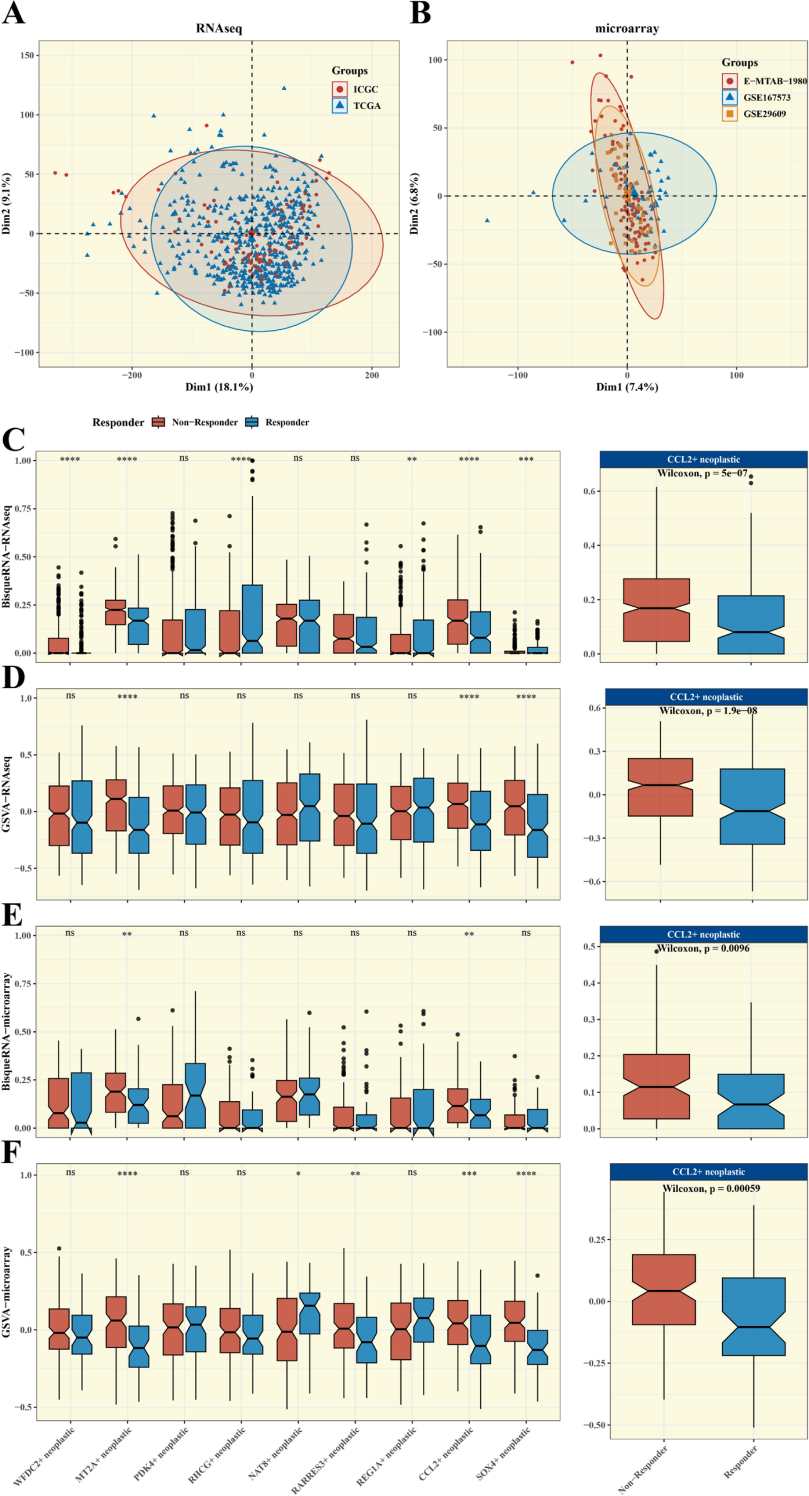
Integrative Analysis with Immune Cohorts. (A, B) PCA plots after batch effect removal for RNAseq (combined TCGA and ICGC) and microarray cohorts. (C–F) Box plots showing differences in the composition/scores of tumour subpopulations in bulk data calculated by BisqueRNA and GSVA algorithms.

We embraced a comprehensive analytical framework that harmoniously integrated RNA sequencing and microarray data, leveraging the TIDE algorithm to foresee the efficacy of immunotherapy in tumours. To uphold the integrity of our findings, we implemented the Combat algorithm, effectively mitigating batch effects that could potentially skew our results across disparate datasets, as exemplified in Figure 3A,B. Furthermore, we leveraged BisqueRNA software to predict the composition of tumour subpopulations in bulk data and assessed the scores of these subpopulations using the GSVA algorithm.

In our examination utilising RNA sequencing data, a notably elevated frequency of CCL2‐expressing malignant cells was detected within the cohort that did not respond to treatment, as opposed to those who did. This finding was supported by a significant *p* value of 5e^−07^ in the BisqueRNA analysis (see Figure 3C), and a similarly significant difference with *p* = 1.9e‐08 was observed in the GSVA analysis (see Figure 3D). Additionally, our analysis using microarray datasets revealed a similar pattern: the concentration of neoplastic cells expressing CCL2 was markedly greater in the group that failed to respond to therapy compared with the group that exhibited a response, with a *p* value of 0.0096 in the BisqueRNA analysis (see Figure 3E) and a highly significant *p* value of 0.00059 in the GSVA analysis (see Figure 3F). In our in vitro qPCR experiments, we also found that the gene expression levels of CCL‐2 and NAT8 were higher in 786‐O cells compared to HK‐2 cells (Figures S2 and S3).

### 3.4 Tumour Subpopulations, Prognosis, and Spatial Transcriptomics Analysis

**FIGURE 4:**
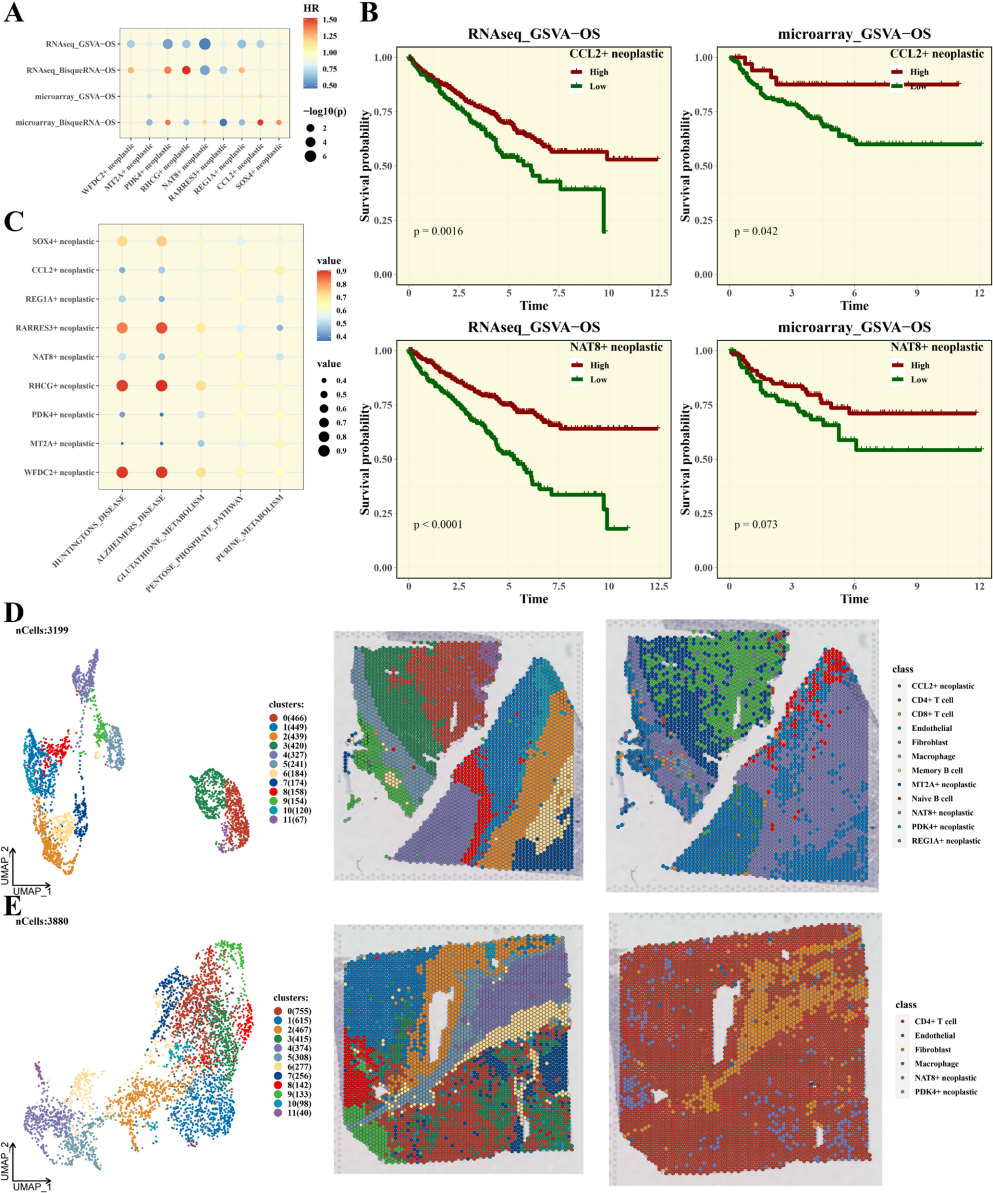
Correlation of Tumour Subpopulations with Prognosis and Empty Droplet Analysis. (A) Bubble plots showing the correlation between composition/scores calculated by BisqueRNA and GSVA in two datasets and prognosis. (B) Survival analysis results based on the composition/scores of CCL2+ neoplastic and NAT8+ neoplastic. (C) Bubble plot displaying the association of tumour cells with functional metabolic pathways. (D, E) Analysis and annotation results of two empty droplet samples.

In this study, we analysed the composition and scores of tumour cell subpopulations in two different datasets using two computational methods: BisqueRNA and GSVA. We explored the correlation between these biomarkers and patient outcomes, with the findings graphically represented through bubble plots, as depicted in Figure 4A. Subsequently, we conducted survival analyses focusing on the composition and scores of CCL2+ neoplastic and NAT8+ neoplastic cell subpopulations. The analysis revealed that patients with higher proportions of CCL2+ neoplastic cell subpopulation in the RNAseq dataset exhibited significantly higher survival rates (*p* = 0.0016), and similarly, patients with higher proportions of NAT8+ neoplastic cell subpopulation also exhibited markedly improved survival outcomes (*p* < 0.0001), as demonstrated in Figure 4B. In the microarray dataset, we observed that patients with higher proportions of CCL2+ neoplastic cell subpopulation had significantly higher survival rates compared to those with lower proportions (*p*=0.042). Associations between tumour cells and functional metabolic pathways were analysed (Figure 4C), and empty droplet samples were annotated, revealing a higher proportion of tumour cells in these samples (Figure 4D,E).

### 3.5 Establishment of a Prognostic Model

**FIGURE 5:**
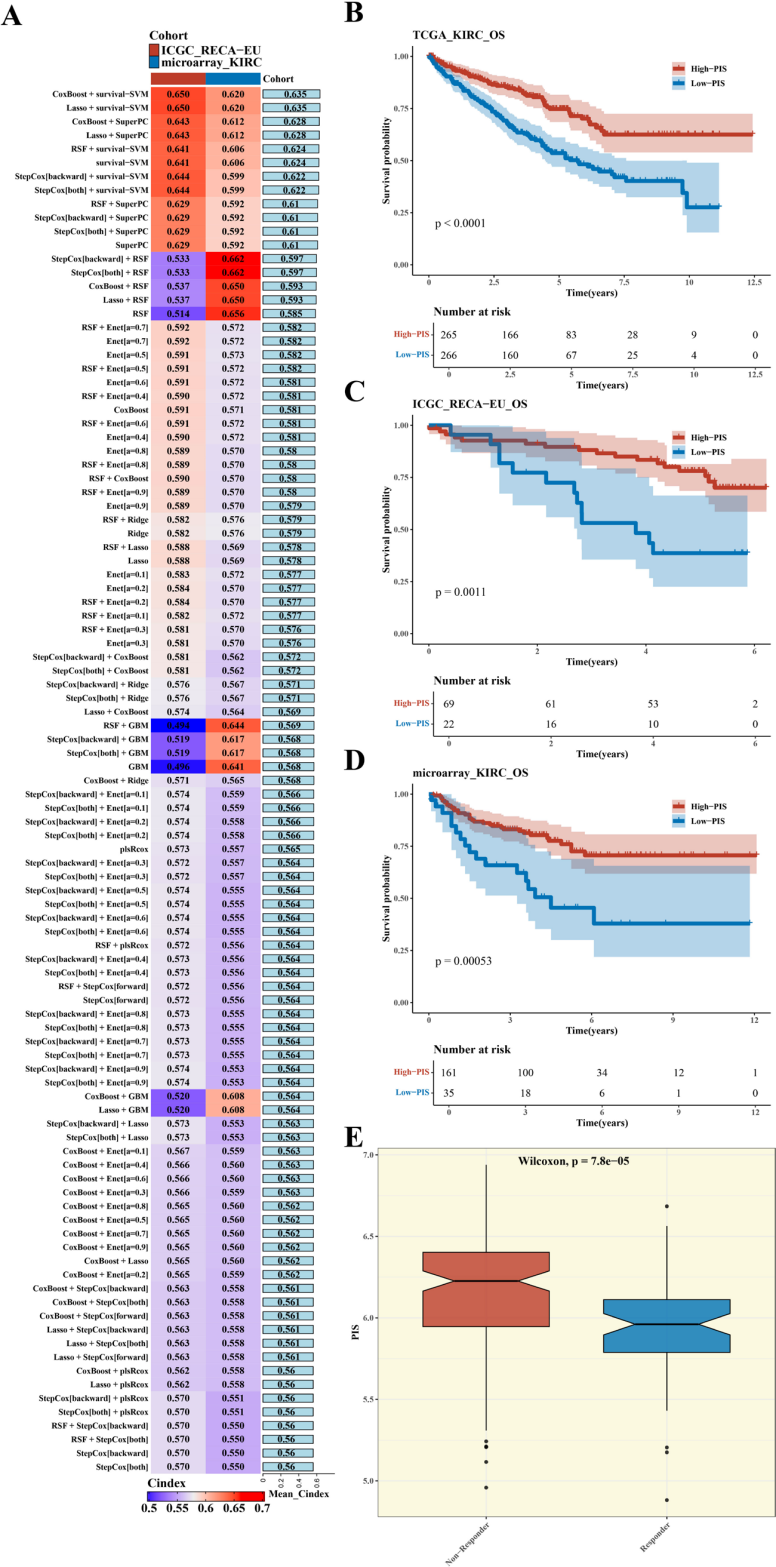
Prognostic Model Establishment. (A) Heatmap of model construction using 101 algorithms. (B–D) Survival analysis results for each cohort. (E) Box plot of PIS in non‐responder and responder groups in the microarray cohort.

In this research, we developed a predictive model for patient outcomes using biomarkers associated with CCL2‐expressing tumour cells, leveraging a combination of data sources and a suite of computational methods. Following thorough examination and verification, we concluded that the fusion of Lasso regression with the survival Support Vector Machine (SVM) emerged as the most effective approach for the development of the predictive model. The model achieved a Concordance Index (C‐index) of 0.650, indicating good discrimination (see Figure 5A).

Advanced survival analyses indicated that individuals with reduced Prognostic Index System (PIS) scores faced inferior outcomes. Within the TCGA KIRC dataset, subjects assigned to the high PIS score cohort demonstrated markedly superior survival probabilities when contrasted with those in the low PIS score cohort (*p* < 0.0001, refer to Figure 5B). This pattern was mirrored in the ICGC RECA‐EU dataset, where individuals with elevated PIS scores recorded significantly enhanced survival probabilities compared to their counterparts with diminished PIS scores (*p* = 0.0011, as shown in Figure 5C), and a comparable trend was noted in the microarray KIRC dataset (*p* = 0.00053, as delineated in Figure 5D).

Moreover, in the microarray cohort, we found that the PIS scores of non‐responding patients were significantly higher than those of responding patients (*p* = 7.8e^−5^, see Figure 5E), suggesting that PIS scores may be associated with patients' response to treatment.

### 3.6 Comparison of Prognostic Models

**FIGURE 6:**
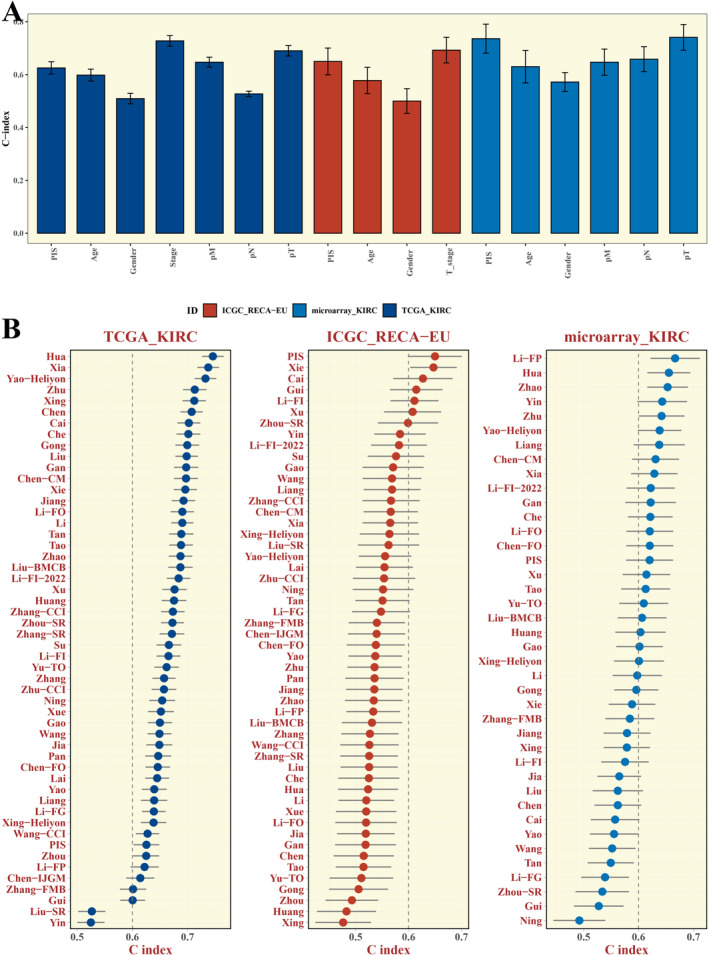
Comparison of Prognostic Models. (A) Bar chart comparing the Cindex of PIS with other clinical indicators in various cohorts. (B) Cindex comparison of PIS with models reported in literature across different cohorts.

The Concordance Index (C‐index) of PIS in comparison to other clinical indicators was analysed, and PIS performance across different cohorts was evaluated (Figure 6A,B) to assess the model's performance.

### 3.7 Mutation Analysis

**FIGURE 7:**
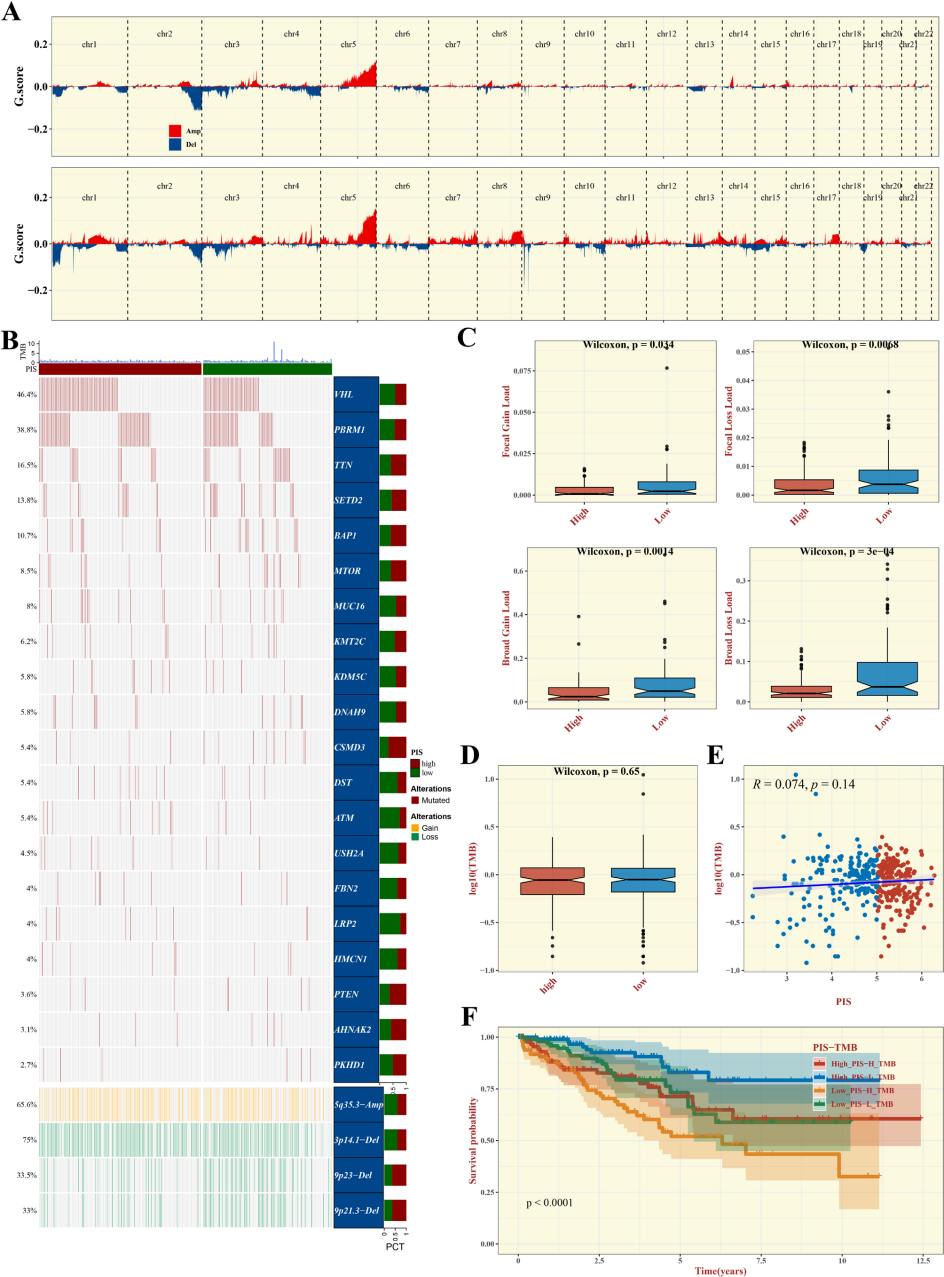
Genomic Alteration Assessment. (A) Graphical representation of CNV disparities between the high and low PIS cohorts. (B) A waterfall diagram juxtaposing mutation and CNV profiles across the two groups. (C–E) Comparative analysis of CNV and TMB within the high and low PIS cohorts. (F) Synergistic survival analysis integrating PIS and TMB metrics.

Discrepancies in CNVs between the high and low PIS score groups were graphically represented (Figure 7A). Subsequently, a waterfall plot was employed to depict the mutations and CNVs across both groups (Figure 7B). Examinations into the disparities of CNVs and TMB between the high and low PIS score groups were conducted (Figure 7C,D). Ultimately, the outcomes of the survival analysis integrating PIS and TMB were showcased (Figure 7E).

### 3.8 Immune Infiltration Analysis

**FIGURE 8:**
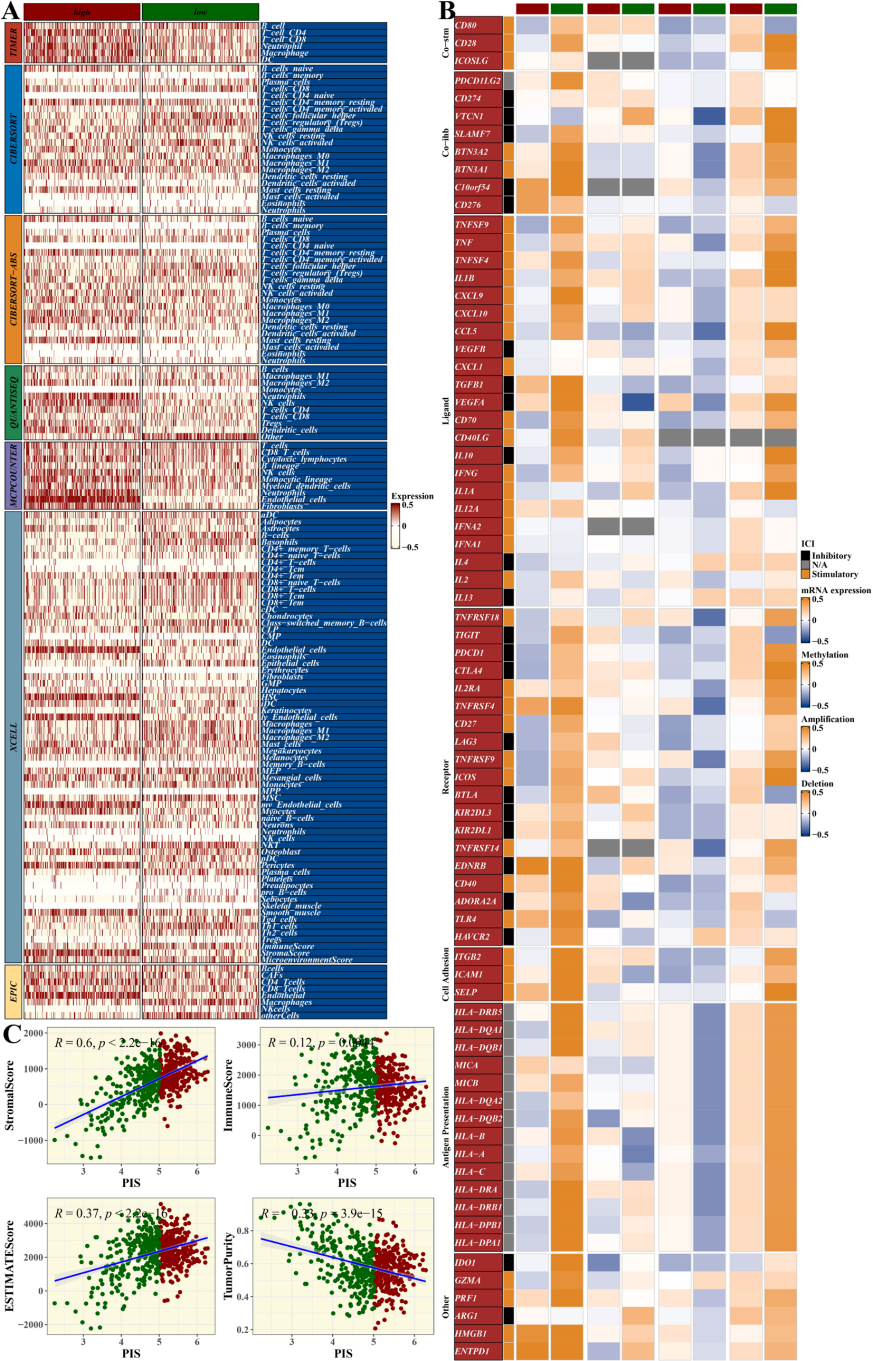
Immune Infiltration Analysis. (A) Heatmap of immune infiltration analysis using seven algorithms from the IOBR package. (B) Heatmap showing the correlation between PIS and the expression, methylation level, CNV amplification and CNV deletion of immune‐related genes. (C) Results of the correlation between four scores calculated by the ESTIMATE algorithm and PIS.

Seven immune infiltration algorithms from the IOBR package were used to estimate immune infiltration in the TCGA dataset, which was displayed in a heatmap (Figure 8A). Correlations between PIS and immune‐related gene expression, methylation levels, CNV amplifications and CNV deletions were calculated and visualised in a heatmap (Figure 8B). Four scores from the ESTIMATE algorithm were correlated with PIS (Figure 8C).

### 3.9 Functional Analysis

**FIGURE 9:**
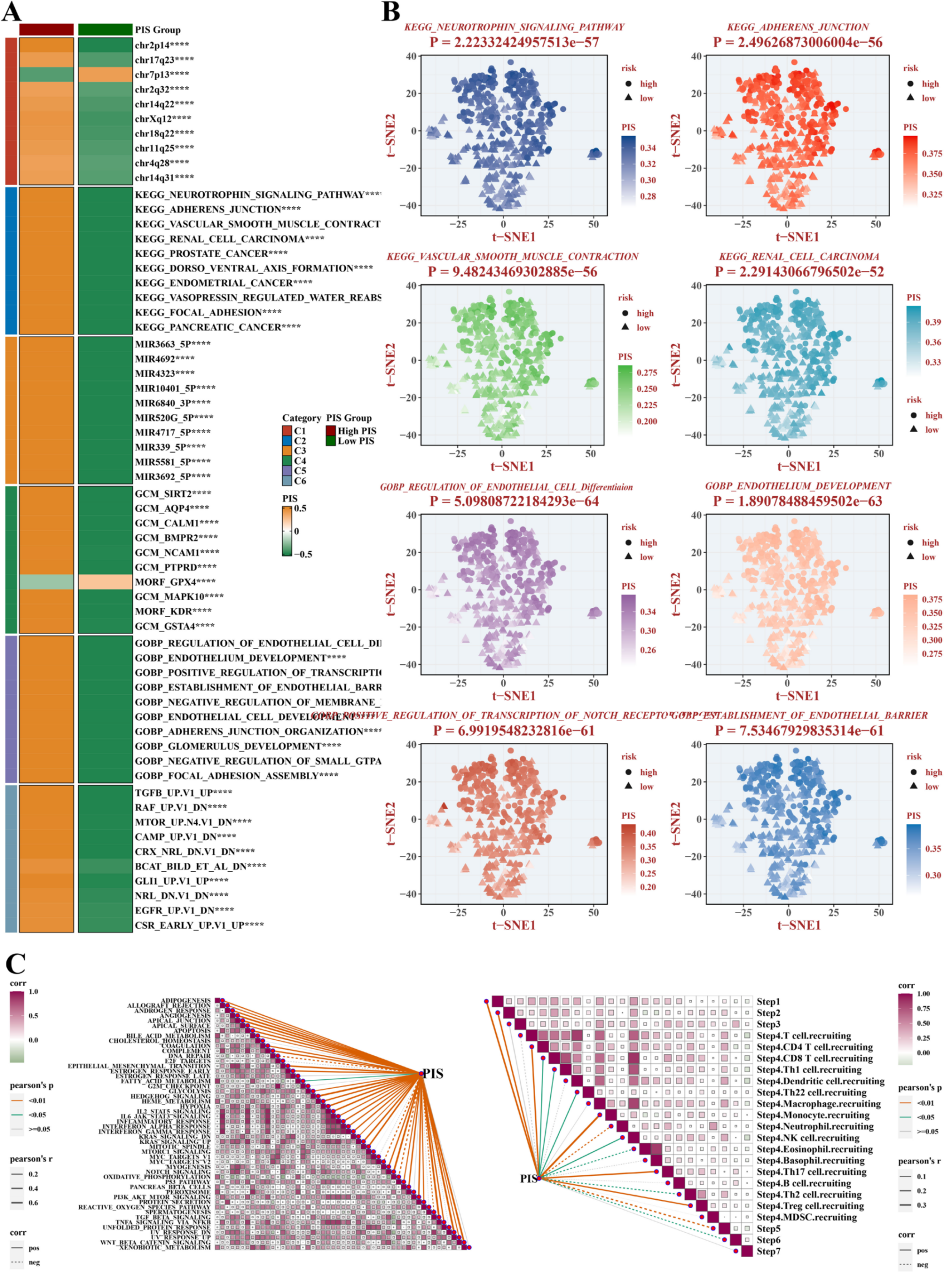
Functional Analysis. (A) Heatmap of ssGSEA scores for functional signatures C1–C6 in each group. (B) tSNE plots of four functional pathways selected from GOBP and KEGG. (C) Correlation plot between PIS and 50 Hallmark pathways and tumour immune‐related functions from TIP data.

In our research, we procured functional signatures spanning categories C1 through C6 from the Msigdb database and quantified these signatures by applying the ssGSEA computational method. From each category, we selected 10 significant pathways and visually presented their distribution through a heatmap (Figure 9A). Additionally, we specifically selected four functional pathways from the Gene Ontology Biological Processes (GOBP) and KEGG databases that were most relevant to our study and visualised their analysis using t‐SNE technology (Figure 9B). The four most relevant pathways from the KEGG database included neurotrophin signalling pathway, focal adhesion, vascular smooth muscle contraction and renal cell carcinoma. Meanwhile, the four most relevant biological functions from GOBP encompassed regulation of endothelial cell differentiation, endothelium development, positive regulation of Notch receptor target gene transcription, and establishment of endothelial barrier. Furthermore, we compiled 50 Hallmark pathways and tumour immune‐related profiles (TIP data) and scored these pathways using the ssGSEA algorithm. We then analysed their correlations with the PIS (Figure 9C). Meanwhile, we also validated the high expression and activation of the Notch pathway in ccRCC through qPCR (Figures [Link], [Link], [Link]).

### 3.10 Analysis of Immunotherapy Response

**FIGURE 10:**
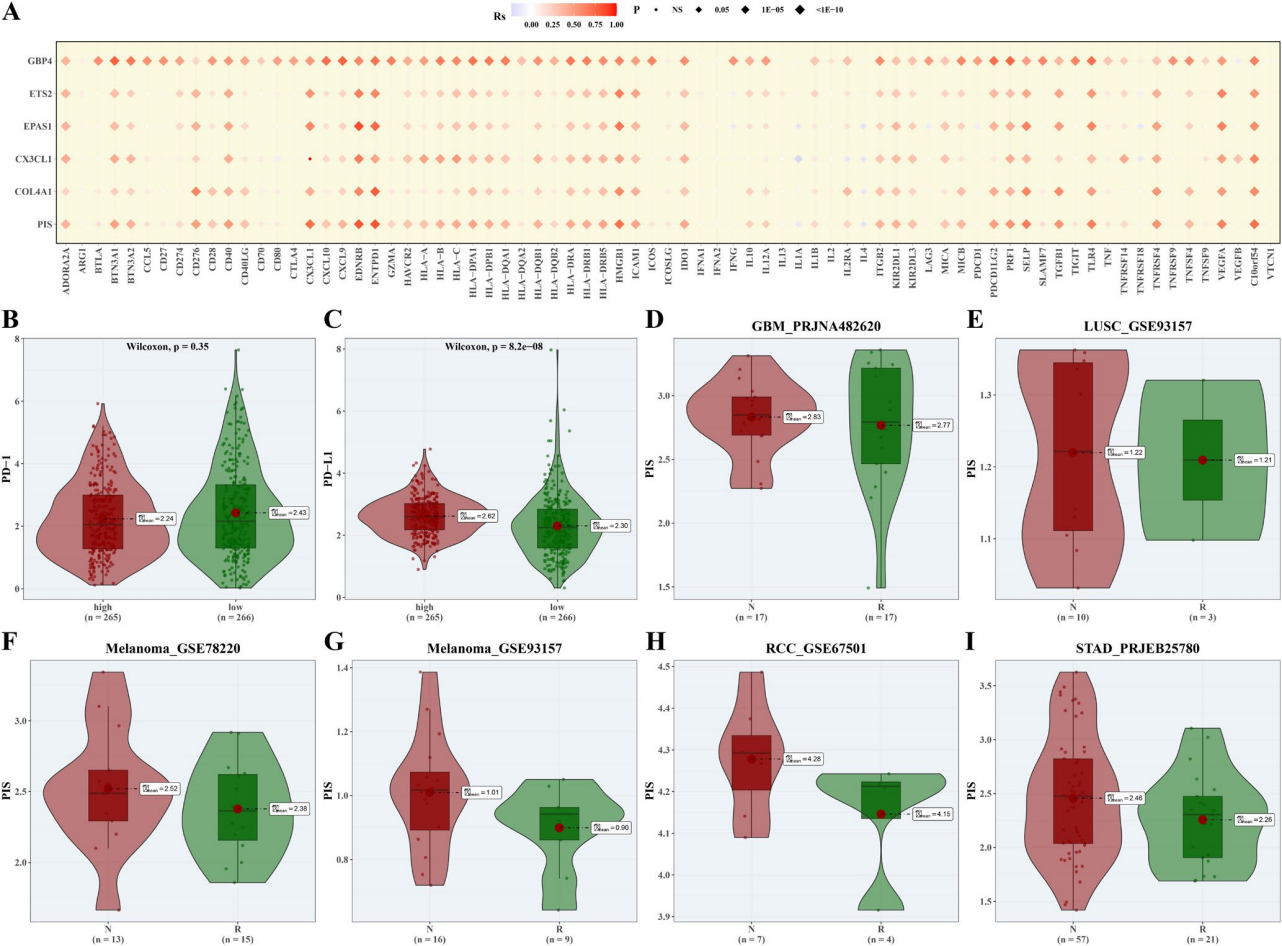
Analysis of Immunotherapy Response. (A) A bubble chart depicting the association among PIS, genes instrumental in model construction, and genes pertinent to immune function. (B, C) Violin diagrams highlighting the variance in the expression of PDCD1 and CD274 across high‐ and low‐risk patient groups. (D–I) A series of violin diagrams portraying the divergence in PIS scores between patients who responded to immunotherapy and those who did not, across six distinct immunotherapy studies.

Correlations between PIS, model‐building genes, and immune‐related genes were analysed, with correlation bubble plots presented (Figure 10A). Discrepancies in the expression levels of PDCD1 (PD‐1) and CD274 (PD‐L1) genes between the groups classified as high‐risk and low‐risk were quantified and illustrated (Figure 10B,C). PIS scores were computed in six immunotherapy cohorts, revealing higher PIS scores in non‐responding groups compared to responding groups (Figure 10D–I).

## 4 Discussion

In this research, we performed an exhaustive examination of single‐cell RNA sequencing data from ccRCC, in conjunction with datasets from immunotherapy studies, to explore the predictive significance of genes within tumours that are associated with responses to immunotherapy in ccRCC. Drawing from these findings, we developed a highly accurate prognostic model.

Our analysis of RNA sequencing and microarray datasets revealed a notable disparity in CCL2+ neoplastic cell prevalence between treatment‐responsive and non‐responsive groups. Specifically, a significantly higher proportion of CCL2+ neoplastic cells was observed in the non‐responsive cohort, implying that this subpopulation may be associated with suboptimal treatment outcomes. We speculate that the elevated presence of CCL2+ neoplastic cells could stem from their augmented survival and proliferation abilities within the TME, ultimately influencing the therapeutic efficacy.

CCL2, a key chemokine, orchestrates intricate functions within the TME. It serves as a magnet, attracting diverse immune cells including regulatory T cells, myeloid‐derived suppressor cells, and monocytes, thereby fostering an immunosuppressive landscape. Beyond immune modulation, CCL2 directly influences tumour angiogenesis and cellular dynamics [27, 28]. In gliomas, CCL2's production is vital for recruiting immunosuppressive CCR4+ Tregs and CCR2^+^ Ly‐6C^+^ monocytic myeloid‐derived suppressor cells [27]. while in breast cancer models, it increases tumour neovascularization by promoting the development and mobilisation of endothelial precursor cells [29]. Furthermore, CCL2 can enhance the tumoricidal capacity of neutrophils [30] and interact with tumour‐associated macrophages, inducing the transition of non‐tumorigenic breast epithelial cells to an invasive phenotype [31]. Notably, in glioblastoma, heightened CCL2 expression aligns with diminished overall patient survival, hinting at its potential as a prognostic biomarker for adverse outcomes [27].

In this study, by integrating multiple datasets and applying a variety of algorithms, we ultimately identified the combination of Lasso regression and survival SVM as the optimal algorithm for constructing the prognostic model. This choice was based on the effectiveness and accuracy of Lasso regression in variable selection for high‐dimensional bioinformatics data, reducing the complexity of the model, and the advantages of survival SVM in handling time‐to‐event data, which provides robust predictive performance.

The model achieved a C‐index of 0.650, indicating a moderate level of discrimination. While this value is not exceptionally high, it still demonstrates the model's capability to distinguish between different risk groups in terms of prognosis. In clinical practice, a C‐index greater than 0.5 signifies that the model has an advantage over random guessing, thus our model shows its potential value in actual clinical applications [32].

Further survival analysis revealed an inverse correlation between patients' PIS and their prognosis: the lower the PIS, the poorer the patient's prognosis. This trend is consistent with research in areas such as breast cancer [33], where prognostic scoring systems are also based on the integrated analysis of gene expression and clinical pathological characteristics. In the TCGA KIRC, ICGC RECA‐EU, and microarray KIRC datasets, patient groups with higher PIS exhibited significantly better survival rates, further validating the effectiveness of our model. Notably, in the microarray cohort, patients who did not respond to treatment had significantly higher PIS scores than those who did, suggesting that PIS may be associated with patients' responsiveness to treatment. This is in line with findings from colorectal cancer research, where a prognostic model based on specific gene signatures not only predicted patient survival probabilities but also supplemented the traditional TNM staging system [32]. In summary, this study successfully constructed an effective prognostic model using a combination of Lasso regression and SVM, ensuring its reliability and practicality through rigorous selection and validation processes, providing valuable references for clinical treatment decisions.

Our investigation pinpointed the neurotrophin signalling pathway, focal adhesion, vascular smooth muscle contraction, and RCC as the most pertinent KEGG pathways. Additionally, the GOBP analysis highlighted the regulation of endothelial cell differentiation, endothelial layer development, positive regulation of Notch receptor target gene transcription, and establishment of endothelial barrier as crucial biological functions underlying our findings.

The Notch signalling pathway has garnered significant attention in the context of ccRCC, underscoring its pivotal role in the onset and progression of this malignancy. A comprehensive analysis of 415 ccRCC patients revealed that 44% harboured genetic alterations within the Notch gene set, with KAT2B and MAML1 exhibiting alterations in 13% and 19% of cases, respectively, both of which are functionally relevant in ccRCC [34]. Notably, a heightened Notch score is associated with an abundance of “resting” or “anti‐inflammatory” tumour‐infiltrating immune cells over their “activated” or “pro‐inflammatory” counterparts, along with the suppression of immune pathways [35]. This finding implies that patients with Notch‐activated ccRCC may experience limited therapeutic benefits from immune checkpoint inhibitors (ICIs). Furthermore, long non‐coding RNAs implicated in the Notch pathway have been linked to unfavourable prognoses and suboptimal responses to immunotherapy or targeted therapy in ccRCC patients [36].

Angiogenesis, a fundamental process underpinning the development and dissemination of ccRCC, is intricately regulated by the Notch signalling pathway. This pathway not only orchestrates normal vascular development but also plays a pivotal role in driving the progression of ccRCC by modulating the angiogenic process [37]. To tackle ccRCC, targeted therapeutic strategies have been devised, focusing on crucial molecular pathways pivotal to endothelial cell differentiation and angiogenesis. These include the VEGF and mTOR pathways. Notably, VEGF TKIs and PD‐1/PD‐L1 immune checkpoint inhibitors have emerged as potent therapeutic options, demonstrating remarkable efficacy in the treatment of ccRCC [38, 39, 40]. VEGF, a pivotal player in fostering tumour angiogenesis, triggers signalling cascades upon binding to its receptors. This activation leads to the formation of new blood vessels, a vital process that sustains tumour growth and survival [41]. In the therapeutic management of metastatic RCC, VEGF inhibitors such as bevacizumab have not only extended patients' progression‐free and overall survival but also shown enhanced effects when used in combination with chemotherapy [42]. Changes in VEGF levels are also considered potential biomarkers for predicting the efficacy of bevacizumab [43]. mTOR, a central intracellular signalling pathway that governs cell growth, metabolism, and autophagy, has exhibited therapeutic promise in metastatic renal cell carcinoma. Notably, the use of mTOR inhibitors, such as temsirolimus, in combination with VEGF inhibitors has shown enhanced efficacy in treating this malignancy [44]. The development of these therapeutic strategies provides more treatment options for ccRCC patients and holds promise for improving patient prognosis.

The quest for prognostic markers continues to gain momentum, with recent studies suggesting that the expression profiles of specific angiogenesis‐associated genes may hold significant predictive value for patient survival and treatment responsiveness [45]. This lays the groundwork for developing new biomarkers that can predict how patients will respond to specific therapies or the prognosis of their disease.

In essence, the influence of the Notch signalling pathway in ccRCC transcends angiogenesis, extending to the regulation of the TME by orchestrating the behaviour of endothelial cells. Targeted therapeutic strategies against this pathway are becoming an integral part of ccRCC treatment. Furthermore, investigating the interplay between the Notch signalling pathway and other signalling pathways will aid in a better understanding of the pathophysiology of ccRCC and the development of more effective treatment strategies.

## 5 Conclusion

The study successfully identified a set of 11 prognostic genes linked to immunotherapy response in ccRCC, and the developed prognostic model, integrating Lasso regression with SVM, showed strong predictive capabilities. Our analysis underscored the paramount significance of the TME in shaping patient prognosis, particularly through the infiltration of CCL2+ neoplastic cells. Furthermore, it emphasised the pivotal role of GOBP‐guided mechanisms in modulating endothelial cell functionalities and the tumour's angiogenic potential.

## 6 Limitations and Future Outlook

While this study offers valuable insights, it has limitations, including the reliance on public databases which may introduce selection bias. For the advancement of our understanding, future endeavours should strive to validate the prognostic model in distinct clinical cohorts and delve deeper into the molecular underpinnings of TME contribution to ccRCC. Additionally, incorporating multi‐omics data could provide a more holistic understanding of ccRCC, potentially leading to the discovery of novel therapeutic targets.

## Author Contributions

**Tongfei Fu:** conceptualization (equal), writing – original draft (equal). **Xinyi Zhang:** data curation (equal), writing – original draft (equal). **Yuedong Liu:** conceptualization (equal), writing – original draft (equal). **Junsong Wu:** data curation (equal), resources (equal). **Xuefeng Liu:** conceptualization (equal), supervision (equal). **Bichao Lu:** formal analysis (equal), supervision (equal). **Yi Huang:** methodology (equal), visualization (equal). **Liping Yang:** data curation (equal), supervision (equal). **Yongli Zhan:** conceptualization (equal), writing – review and editing (equal).

## Conflicts of Interest

The authors declare no conflicts of interest.

## Supporting information


**Figure S1.** UMAP Plot of Cell Annotation Results from Renal Cancer Single‐Cell Data.

**Figure S2.** Expression of CCL2 was validated by RT‐qPCR assay. *** represents *p* < 0.001.

**Figure S3.** Expression of NAT8 was validated by RT‐qPCR assay. *** represents *p* < 0.001.

**Figure S4.** Expression of MAML1 was validated by RT‐qPCR assay. **** represents *p* < 0.0001.

**Figure S5.** Expression of KAT2B was validated by RT‐qPCR assay. *** represents *p* < 0.001.

**Figure S6.** Expression of Notch1 was validated by RT‐qPCR assay. *** represents *p* < 0.001.

**Table S1.** Primer sequences.

## Data Availability

Data available on request from the authors.
