# Supplementary figures and images for: Exploring the Prognostic Value of Tumour‐Associated Genes in Clear Cell Renal Cell Carcinoma Through Single‐Cell RNA Sequencing Insights
Source: J Cell Mol Med. 2024 Dec 20;28(24):e70297. doi: 10.1111/jcmm.70297 (PMC11661917; doi:10.1111/jcmm.70297)

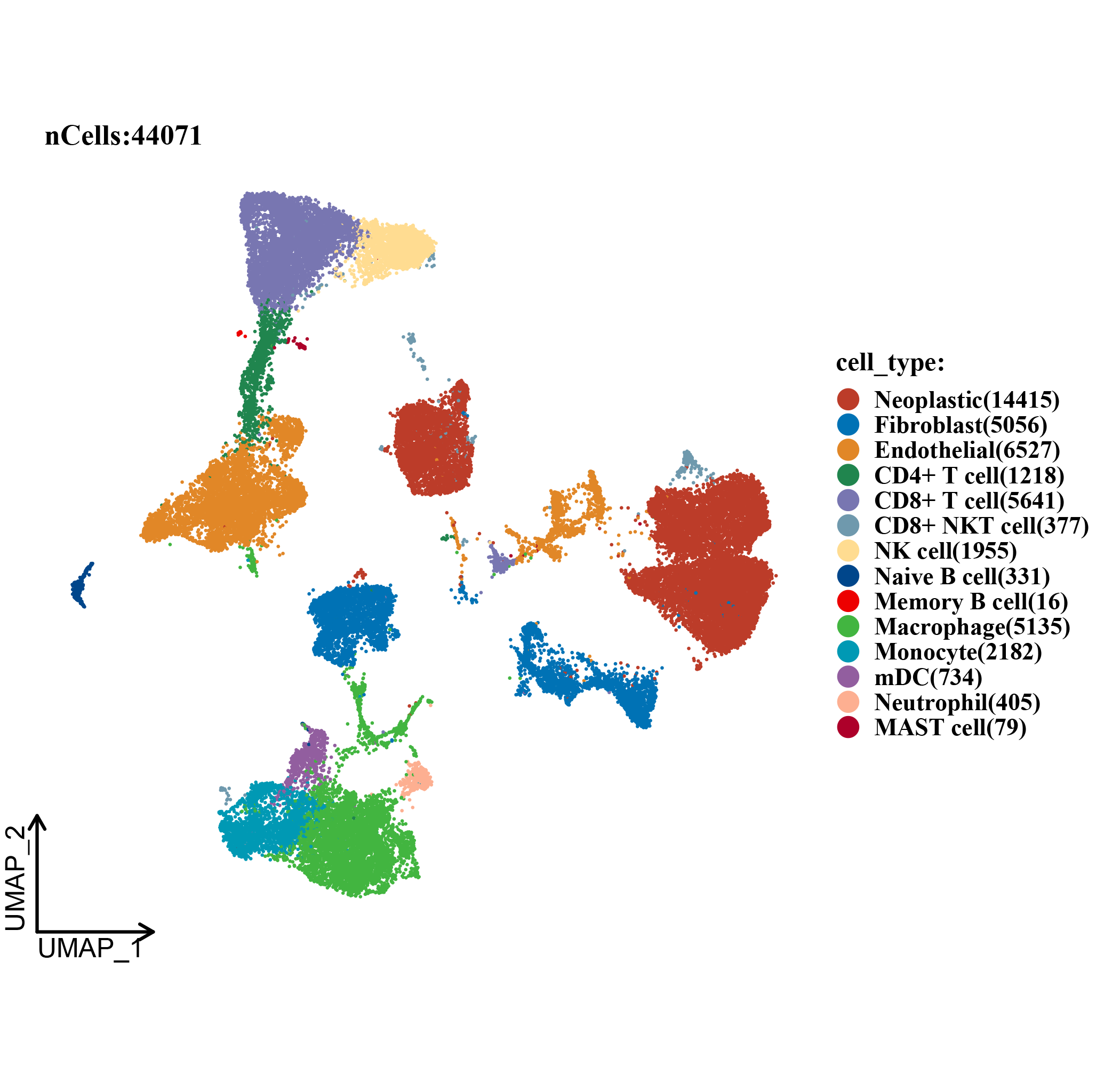

Supplement: Supplementary file 1 — Figure S1. UMAP Plot of Cell Annotation Results from Renal Cancer Single‐Cell Data. [file JCMM-28-e70297-s002.tif]

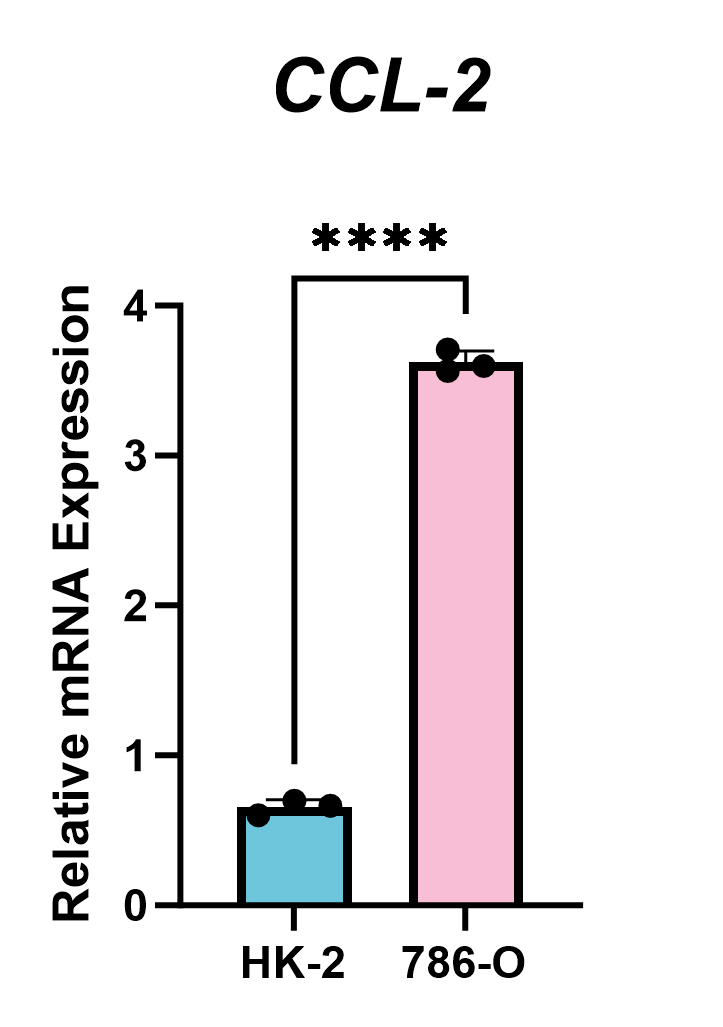

Supplement: Supplementary file 2 — Figure S2. Expression of CCL2 was validated by RT‐qPCR assay. *** represents p < 0.001. [file JCMM-28-e70297-s003.tif]

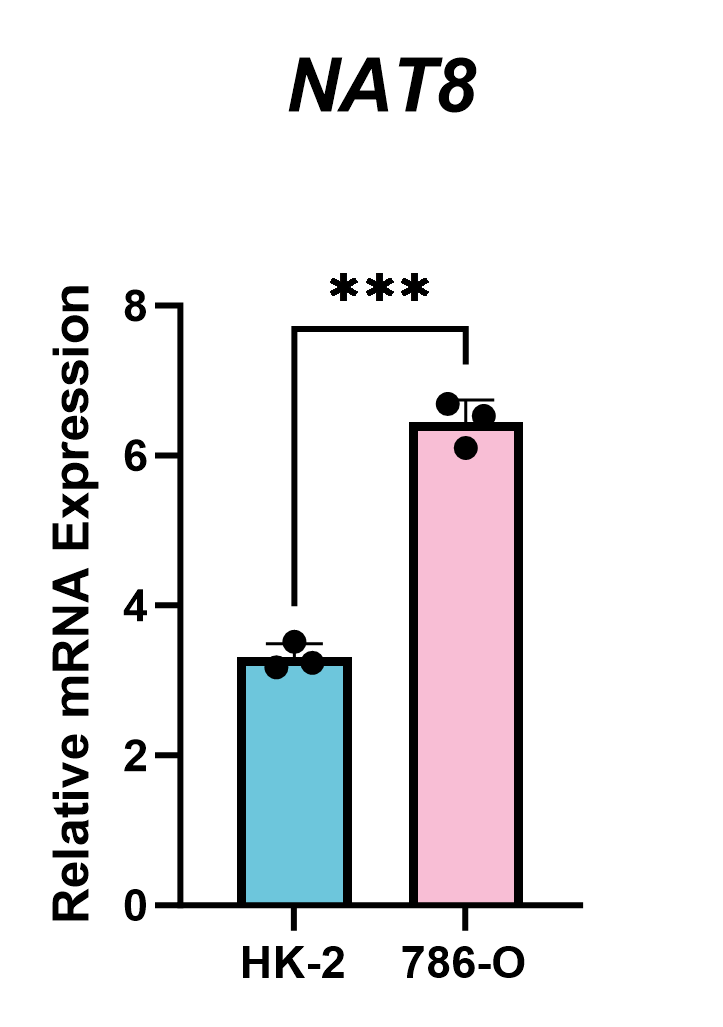

Supplement: Supplementary file 3 — Figure S3. Expression of NAT8 was validated by RT‐qPCR assay. *** represents p < 0.001. [file JCMM-28-e70297-s005.tif]

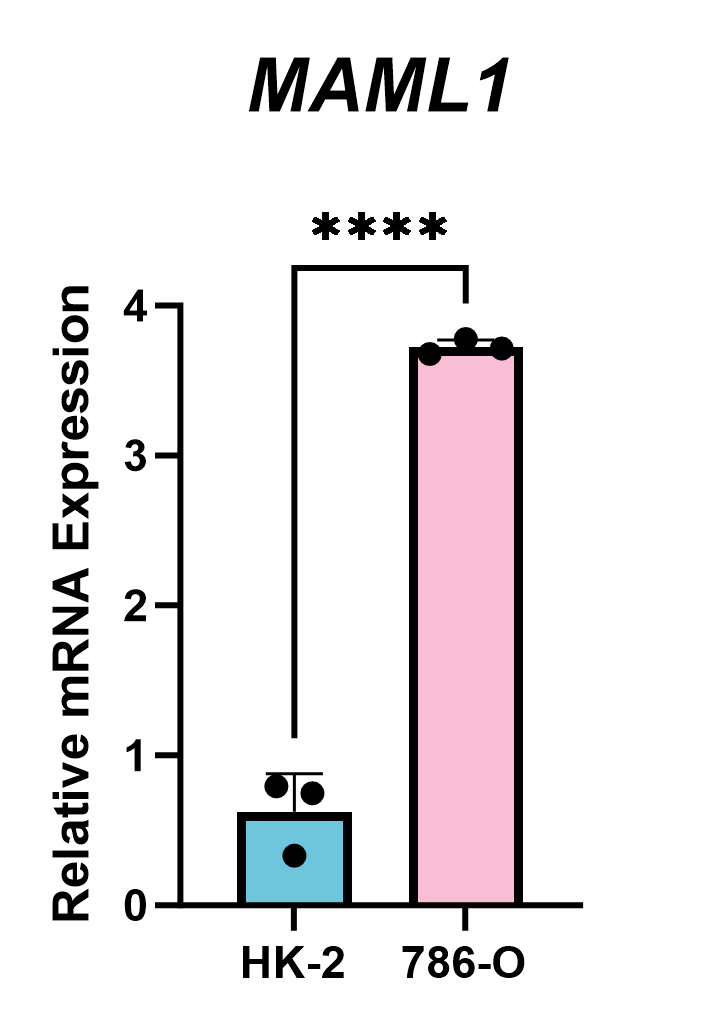

Supplement: Supplementary file 4 — Figure S4. Expression of MAML1 was validated by RT‐qPCR assay. **** represents p < 0.0001. [file JCMM-28-e70297-s004.tif]

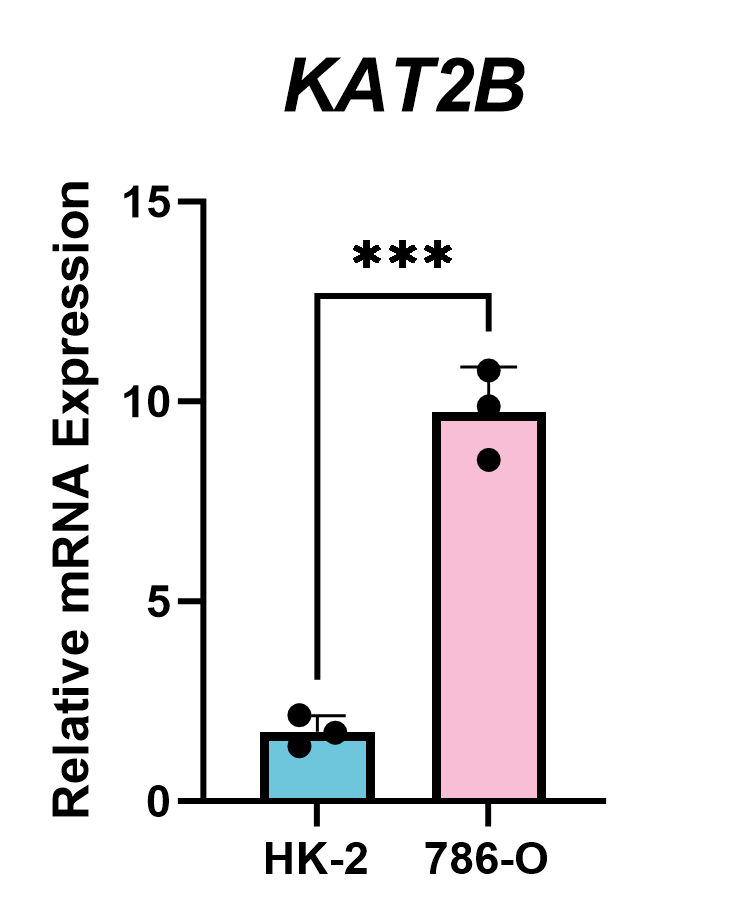

Supplement: Supplementary file 5 — Figure S5. Expression of KAT2B was validated by RT‐qPCR assay. *** represents p < 0.001. [file JCMM-28-e70297-s006.tif]

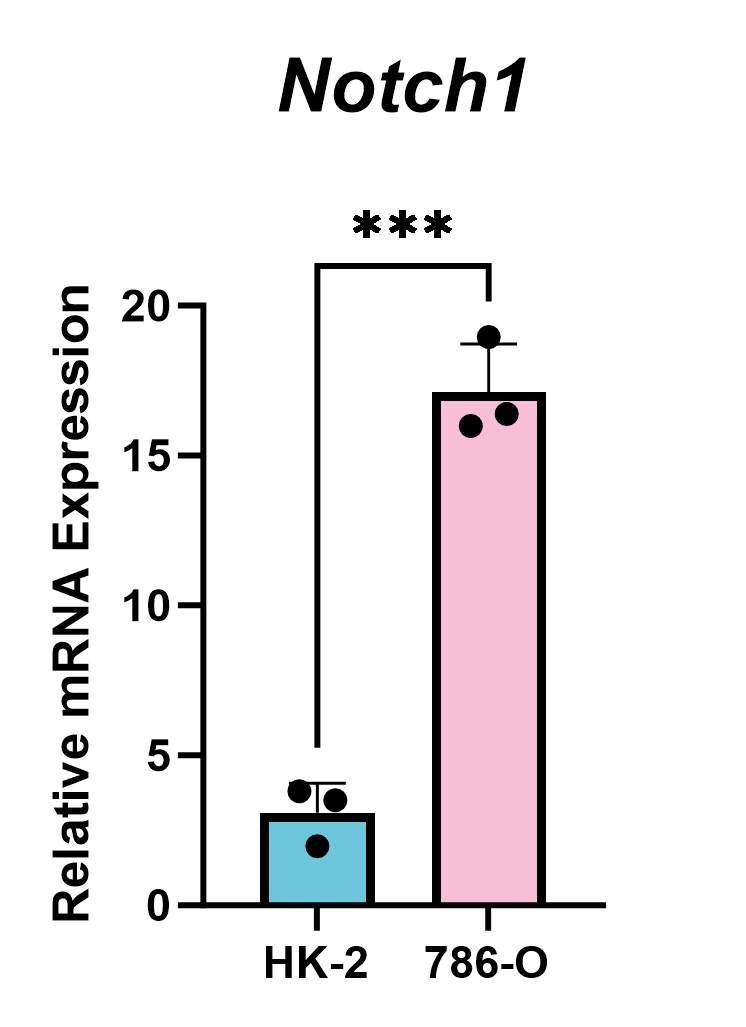

Supplement: Supplementary file 6 — Figure S6. Expression of Notch1 was validated by RT‐qPCR assay. *** represents p < 0.001. [file JCMM-28-e70297-s007.tif]
